# A Potential Route to Reduce Ischemia/Reperfusion Injury in Organ Preservation

**DOI:** 10.3390/cells11172763

**Published:** 2022-09-05

**Authors:** Marc Micó-Carnero, Mohamed Amine Zaouali, Carlos Rojano-Alfonso, Cristina Maroto-Serrat, Hassen Ben Abdennebi, Carmen Peralta

**Affiliations:** 1 Institut of Biomedical Research August Pi i Sunyer (IDIBAPS), 08036 Barcelona, Spain; 2 Laboratory of Human Genome and Multifactorial Diseases (LR12ES07), Faculty of Pharmacy, University of Monastir, Monastir 5000, Tunisia

**Keywords:** ischemia reperfusion injury, IRI, organ transplantation, cold storage solution, organ preservation, steatosis, ECD grafts, trimetazidine, carvedilol, tacrolimus

## Abstract

The pathophysiological process of ischemia and reperfusion injury (IRI), an inevitable step in organ transplantation, causes important biochemical and structural changes that can result in serious organ damage. IRI is relevant for early graft dysfunction and graft survival. Today, in a global context of organ shortages, most organs come from extended criteria donors (ECDs), which are more sensitive to IRI. The main objective of organ preservation solutions is to protect against IRI through the application of specific, nonphysiological components, under conditions of no blood or oxygen, and then under conditions of metabolic reduction by hypothermia. The composition of hypothermic solutions includes osmotic and oncotic buffering components, and they are intracellular (rich in potassium) or extracellular (rich in sodium). However, above all, they all contain the same type of components intended to protect against IRI, such as glutathione, adenosine and allopurinol. These components have not changed for more than 30 years, even though our knowledge of IRI, and much of the relevant literature, questions their stability or efficacy. In addition, several pharmacological molecules have been the subjects of preclinical studies to optimize this protection. Among them, trimetazidine, tacrolimus and carvedilol have shown the most benefits. In fact, these drugs are already in clinical use, and it is a question of repositioning them for this novel use, without additional risk. This new strategy of including them would allow us to shift from cold storage solutions to cold preservation solutions including multitarget pharmacological components, offering protection against IRI and thus protecting today’s more vulnerable organs.

## 1. Introduction

Nowadays, organ transplantation provides the best available solution and is the clinically accepted treatment for end-stage organ failure including end-stage renal disease (ESRD) and liver failure [1]. In this context, the goal of organ preservation is to maintain grafts in viable conditions outside the body, while being transferred from the donor to the recipient. This step inevitably leads to the pathophysiological process of ischemia/reperfusion injury (IRI) [2].

IRI has been shown to be an important contributor to early graft dysfunction (EGD), specifically to renal delayed graft function and early allograft dysfunction in liver [3,4,5,6]. In addition, this initial EGD has a negative impact on long-term survival [7,8]. Meanwhile, the increasing number of patients on waiting lists for organ transplantation has obliged transplant teams to consider organs from so-called extended criteria donors (ECDs). However, such organs present increased risk of EGD after transplantation due to their heightened vulnerability to IRI [9,10,11,12,13].

The currently accepted standard method for the preservation of allografts worldwide is cold storage. This method has the enormous benefit of simplicity. However, it involves a phase of ischemia, since from organ procurement, during transport and until transplantation into the recipient, the organ is inevitably deprived of blood, which otherwise would provide oxygen and nutrients. To protect an organ during this phase, its metabolism is slowed by flushing and conditioning it with a cold solution at +4 °C, called a cold storage solution (CSS), and it is transported in a hypothermic box. Recently, perfusion machines have appeared on the market, which replace the simple hypothermic box and perfuse the organ with an organ preservation solution at +4 °C, in this case called machine perfusion solution (MPS). Both techniques are used in clinical practice, and in both cases, the organ is in contact with a cold solution [14,15,16,17,18].

After the ischemic phase, the organ is rinsed with a physiological solution, or blood, prior to implantation in the recipient. Graft damage occurs gradually during ischemia with further deterioration taking place during reperfusion after revascularization. During the organ preservation process, the first level of protection is provided by hypothermia, since this reduces the metabolic requirements of the graft.

The solutions used for cold storage or machine perfusion are the vectors by which hypothermia is applied. Indeed, their composition plays a central role in optimizing the quality of the preserved organs and inhibiting or preventing IRI.

In 1988, Belzer et al. defined the concept of abdominal cold storage solution for kidneys, liver and pancreas and protection against IRI [19]. These authors showed that the solutions employed must provide a physical and chemical environment (ions and substrates) that limit cellular and interstitial edema and other structural changes during ischemia. The solutions also provide a protective environment that includes components to prevent injury and ensure that the organ is in the best possible condition to restore normal cell metabolism upon reperfusion and organ function after transplantation.

**Figure 1:**
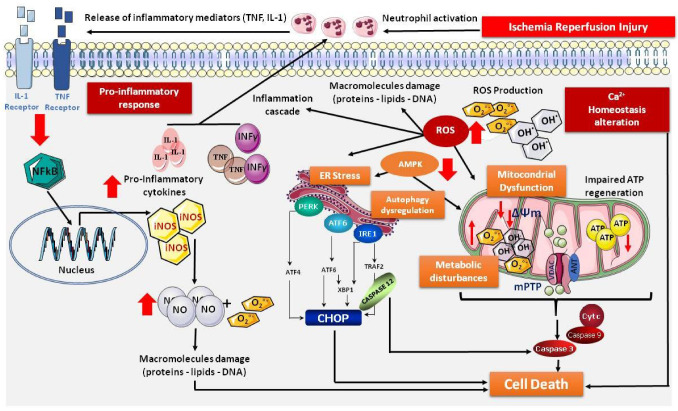
Schematic representation of the signaling pathways affected by IRI during organ preservation. ↓: decrease; ↑: increase; AMPK: AMP-activated protein kinase; ANT: adenine nucleotide translocase; ATF: activating transcription factor; ATG7: autophagy-related gene 7; ATP: adenosine triphosphate; Ca^2+^: calcium ion; CHOP: C/EBP homologous protein; CytC: cytrochrome C; ER: endoplasmic reticulum; FKBP: FK506-binding protein; INFγ: interferon-gamma; iNOS: inducible nitric oxide synthase; IL1: interleukin-1; IRE1: inositol-requiring enzyme 1; LC3B: light chain 3 B; mPTP: mitochondrial permeability transition pore; NF-AT: nuclear factor of activated T-cells; NF-κB: nuclear factor kappa-light-chain-enhancer of activated B cells; NO: nitric oxide; O_2_^°−^ ion: superoxide ion; OH^-^: hydroxide ion; PERK: protein kinase RNA-like ER kinase; ROS: reactive oxygen species; TNF: tumor necrosis factor; VDAC: voltage-dependent anionic channel.

IRI causes important biochemical and structural changes that can result in serious organ damage. It is well known that IRI is multifactorial and involves several mediators, and all the organelles of cells are affected by the process, including mitochondria and the endoplasmic reticulum (ER) [20]. Indeed, the reintroduction of oxygenated blood into an ischemic organ, while necessary for the restoration of aerobic ATP production, results in exacerbated oxidative stress and inflammatory responses [21,22]. These pathologic events induce the opening of the mitochondrial permeability transition pore (mPTP) and ER stress. Moreover, IRI produces damage that is closely linked to the triggering of harmful cascades of nearly simultaneous events during the ischemia/reperfusion (I/R) process [2,23,24,25]. Figure 1 is an illustration of this process.

Furthermore, IRI results in a cascade of reactions linked to the exponential generation of highly reactive oxygen species (ROS) and AMPK dysregulation. ROS react with all cell structures, causing damage to proteins, lipids and DNA. In addition, ROS are key in the impairment of mitochondrial and ER function. The stressed ER response activates three sensors: inositol-requiring enzyme 1 (IRE1), protein kinase R-like endoplasmic reticulum kinase (PERK) and activating transcription factor 6 (ATF6). These work as ER signal transducers and exacerbate C/EBP homologous protein (CHOP) transcription via several pathways, thereby promoting cell death. Mitochondrial damage triggers the opening of the mPTP and the release of cytochrome C via proapoptotic proteins promoting cell death. Meanwhile, caspase 12 activation may also result in the stimulation of caspase 3. ROS also promote the synthesis of proinflammatory molecules [21,22,23]. 

Finally, IRI leads to the accumulation of neutrophils, which results in the release of tumor necrosis factor (TNFα) and interleukin 1 (IL-1). In the same way, the binding of IL-1 and TNFα to their respective receptors activates nuclear factor-kappa B (NF-κB), which amplifies the inflammatory response and induces the expression of inducible nitric oxide synthase (iNOS). Large amounts of nitric oxide (NO) are then produced. This NO can interact with the superoxide anion (O_2_^°−^) producing peroxynitrite (ONOO^−^), which is a potent radical that can cause lipid peroxidation [21,22,23].

## 2. Hypothermic Solutions: Potential and Limitations

The main objectives of hypothermic solutions, whether CCS or MPS, are to protect the organ by cooling it and decreasing its metabolism and, above all, to protect it from IRI. The most commonly used CSS compositions for abdominal organs are (in the order of their introduction into clinical use): HTK (histidine–tryptophan–ketoglutarate), Belzer UW (University of Wisconsin), Celsior and IGL-1. The most commonly used hypothermic perfusion solution is Belzer UW Machine Perfusion Solution (MPS). Table 1 shows the composition of the solutions.

Belzer et al. define the components necessary for organ cold storage, considering the pathophysiological situation and IRI [19,26]. Thus, the UW solution contains osmotic agents other than glucose, such as raffinose or lactobionate, which are not metabolized or degraded when the organ is in hypothermia. In addition to a buffer, such as phosphate ions, the UW solution contains an oncotic agent, hydroxyethyl starch (HES), which keeps the liquid in the vascular compartment and thus prevents edema. Moreover, the UW solution includes in its composition agents that protect against IRI, such as glutathione, adenosine and allopurinol. Glutathione was introduced into the UW solution in its reduced form (GSH) as a free radical scavenger and to compensate for ischemia-induced cellular losses of this antioxidant. Adenosine was introduced as a precursor for ATP synthesis; allopurinol was introduced as a xanthine oxidase (XO) inhibitor to reduce the formation of reactive oxygen species (ROS).

Belzer’s solution has remained the gold standard in transplantation of abdominal organs since 1990, as no other composition has demonstrated clinical superiority [27,28,29]. 

Solutions have, however, been modified to improve their ionic composition, such as by incorporating sodium instead of potassium. Celsior, IGL-1 or MPS solutions are of clinical interest because they are extracellular solutions with a high Na^+^ concentration. These high Na^+^ present no clinical risks if there are traces of K^+^ remaining after final organ flushing that pass into circulation in the recipient. They also decrease vasoconstriction for better cooling of the organ, and they preserve ATPase pump function, which improves functional recovery at the moment of reperfusion.

In fact, if there is a high content of K^+^, there is a risk of a remnant of the K^+^ passing into the recipient, which can cause plasma membrane depolarization and vasoconstriction, leading to increased vascular resistance and decreased blood flow during wash-out [30,31,32,33]. In addition, a K^+^-rich composition disrupts electrolyte homeostasis, resulting in accelerated pumping function of the Na^+^/K^+^ ATPase, which maintains ion concentration gradients and consequently induces depletion of ATP stores more rapidly [31]

Later, in 2005, IGL-1 solution replaced HES with polyethylene glycol 35,000 (PEG). HES has been shown to increase the aggregation of red blood cells, resulting in a loss of their physiological function with the consequent risk of impaired wash-out and cooling of the graft [34,35]. In contrast, PEG has been shown not to increase red blood cell aggregation [36,37]. Additionally, it is now well established that PEG exerts oncotic pressure thus reducing cell edema and mitochondrial injury [38,39,40,41,42,43,44]. It has also been found that it reduces cell swelling and protects the cytoskeleton from the effects of hypothermia. Antioxidant properties have been described for different varieties of PEG, specifically those with a high molecular weight [45]. Moreover, PEG has been shown to provide better protection for steatotic livers than for nonsteatotic ones. Ben Mosbah et al. demonstrated that the beneficial effects of IGL-1 solution are through the NO, because they were abolished by the addition of L-NAME [46,47].

Moreover, adding to the composition energy-linked nutrients/metabolites could be considered to counteract I/R-induced dysregulation of enzymes related to carbohydrate, protein and lipid supply, and metabolism could be considered. Furthermore, improvements in clinical temperature control protocols during utilization should be considered to ensure good organ hypothermia throughout the transport phase. This would be a way to optimize further the preservation.

Notwithstanding these adjustments, in the last 30 years, the components that offer protection against IRI have not been changed in CSS or MPS. These include reduced glutathione (GSH), adenosine and allopurinol. However, the efficacy of these protective components has been challenged and questioned.

GSH is unstable and labile and spontaneously oxidizes into oxidized glutathione (GSSG), thereby losing its protective effect [48,49,50,51]. The concentration of GSH in the solutions thus decreases considerably during storage due to this oxidization. Some reports even advise that “fresh” GSH should be added before bringing the solution into contact with the graft. Taking all these observations into account, the protective effect of glutathione remains controversial.

Adenosine cannot maintain ATP homeostasis during ischemia. Indeed, UW solutions from which adenosine was omitted were shown to have similar or even greater benefits during cold liver storage [24,52]. Negative effects of adenosine at the systemic level have also been reported in clinical practice [53].

Finally, studies performed in humans indicate that allopurinol is unable to reduce ROS formation from xanthine/xanthine oxidase during reperfusion [24,54]

## 3. New Strategy to Protect Grafts from Ischemia/Reperfusion Injury

Much effort is being made to discover the ideal combination of agents to be included in preservation solutions to minimize or prevent all the consequences of IRI. Several molecules have been found to be active under cold and warm ischemic conditions [22,55,56,57,58].

In what follows, we focus on trimetazidine, tacrolimus and carvedilol, which present no clinical risks and therefore are an excellent benefit/risk relation for future clinical use. We describe these drugs and their intended action as it stands today, together with the various preclinical research studies that demonstrate their potential with regard to IRI.

This preclinical research is of great importance when it comes to improving organ preservation. As a matter of fact, it is about repositioning these safe drugs into a new application, such as protection against IRI in organ preservation. 

### 3.1. Trimetazidine

Trimetazidine, 1-(2,3,4-trimethoxybenzyl) piperazine, is a cytoprotective agent that has been shown to have beneficial effects in the treatment of angina pectoris and coronary artery diseases in clinical conditions [59,60]. It has been studied in several preclinical in vitro, ex vivo and in vivo models of organ preservation. The data indicate that trimetazidine acts on mitochondria, preserving their metabolic functions, integrity and ionic permeability, as well as reducing oxidative stress [61,62]. This is corroborated by a reduction in xanthine oxidase (XO) activity, which catalyzes the catabolism of purines (AMP) to hypoxanthine and uric acid [63]. In addition, trimetazidine inhibits the β-oxidation of fatty acids in mitochondria under ischemic conditions [64,65]. Salducci et al. observed that trimetazidine restores ATP synthesis in isolated mitochondria previously exposed to calcium overload [66]. Meanwhile, Domanski et al. showed that it improves energy status after renal ischemia/reperfusion (I/R) in rats [67]. In addition, trimetazidine was found to activate eNOS and NO production [67,68,69,70,71]. Moreover, trimetazidine is known to act as a potent antioxidant in different organs [68,72,73,74,75]. Ruixing et al. reported that it provokes a reduction in MDA levels as well as an increase in superoxide dismutase (SOD) activity after I/R [76].

Trimetazidine also decreases cell death. Indeed, it has been shown to have mitochondrial binding sites and to decrease the opening of the mitochondrial permeability transition pore (mPTP) involved in the occurrence of apoptosis [77]. Furthermore, it inhibits glycogen synthase kinase-3 beta (GSK3-β) activity in rats treated with it before ischemia [78]. This activity correlates with decreased voltage-dependent anion channel (VDAC) phosphorylation and decreased apoptosis. 

Trimetazidine exerts part of its protective effect through the induction of SIRT1 protein expression and activity [79,80]. Sirtuins (SIRTs) are enzymes that belong to the family of nicotinamide adenine dinucleotide (NAD)-dependent histone deacetylases and ADP-ribosyl transferases. They are involved in the regulation of many processes, such as inflammation, energy restriction, mitochondrial function, stress resistance, endothelial function and apoptosis [81,82]. SIRT1 has been associated with the pathophysiology of I/R in several organs [79]. Pantazi et al. demonstrated that SIRT1 reduces liver injury and oxidative stress and promotes the activation of AMPK, Hsp70 and autophagy parameters [79,80]. These results were confirmed by Zaouali et al., who observed that the action of trimetazidine on SIRT1 is matched by the inhibition of HMGB1 (high-mobility group box 1: a protein involved in inflammatory processes) and reduction of TNFα and inflammation [83].

In kidney preservation, the use of trimetazidine as an additive to CSS is of interest due to its potential protection against IRI. Faure et al. observed that the addition of trimetazidine to Euro–Collins or UW CSS improves renal graft function in pigs [84,85]. The same result has been reported by other authors using different experimental models [61,62,86,87,88,89,90]. All these studies have shown that the addition of trimetazidine to CSS improves the functional parameters and proteinuria of transplanted animals, while decreasing oxidative stress. Furthermore, trimetazidine maintains the integrity of mitochondria even after 48 h of cold preservation. It reduces the expression of MHC class II molecules and mononuclear cell infiltrates, as well as decreasing the occurrence of interstitial fibrosis, the number of CD4+ and CD8+ infiltrating cells and the expression of VCAM-1 in transplanted kidneys [91]

Regarding liver preservation, Ben Mosbah et al. showed that the addition of trimetazidine to UW solution improves the quality of both steatotic and nonsteatotic liver grafts [71]. Trimetazidine also decreases cytolysis and vascular resistance and increases tissue stores of ATP. This effect is related to the maintenance of mitochondrial function through the inhibition of mPTP opening in preserved livers [69]. It induces the generation of nitric oxide (NO) via the phosphorylation of endothelial nitic oxide synthase (eNOS), depending on the activation of 5’ AMP-activated protein kinase (AMP kinase) [92]. Moreover, the same authors observed that the inhibition of AMP kinase activation by araA and of NO production by NAME abolishes the protective effect of trimetazidine. Thus, the NO generation improves the oxygenation of liver tissue, protects against alteration of hepatic microcirculation and reduces the production of hepatic endothelin. The effect of trimetazidine on NO production during cold ischemia also promotes the action of hypoxia-inducible factor 1 (HIF-1) [93].

### 3.2. Tacrolimus

Tacrolimus, also known as FK506, is a powerful immunosuppressant that has been approved for clinical use in Europe and the USA. It is primarily used in organ transplantation to prevent allograft rejection [94]. Since 1989, the characteristics and mechanisms of action of tacrolimus have been extensively reported [95]. Its action involves the inhibition of lymphocyte activation by blocking IL-2 synthesis. It is a calcineurin inhibitor capable of suppressing in vitro proliferation of activated lymphocytes at a concentration 100 times lower than cyclosporine A [96,97,98]. Tacrolimus is a highly lipophilic molecule that easily crosses the plasma membrane to reach the cytosol. Therefore, its mechanism is not dependent on binding to any cell surface receptor [99]. Within the cell, it binds to a soluble immunophilin called FKBP (FK-binding protein) [95]. The tacrolimus–FKBP complex exhibits a high affinity for linking with the calcineurin–calmodulin complex, thereby inhibiting the calcium-dependent phosphorylation of a potent intranuclear transcriptional regulatory factor known as the nuclear factor of activated T-cells (NF-AT) [100]. The inhibition of NF-AT blocks transcription of the gene encoding IL-2, which in turn inhibits T-cell activation [97,98]. There are other transcription factors, such as AP-1, AP-3, Oct-1 and NF-κB, that calcineurin has the capacity to activate and which can be inhibited by tacrolimus [101,102,103].

Moreover, apart from its immunosuppressive qualities, several studies since 1998 have shown that tacrolimus protects cells against IRI [104]. In 2003, St Peter et al. reviewed the literature to show how tacrolimus interacts with the intricate cellular mechanisms initiated by ischemia and reperfusion [105]. The effects depend on its multiple actions, including the preservation of microcirculation, attenuation of oxidative stress and inhibition of Ca^2+^-dependent intracellular signaling pathways [106,107]. For example, tacrolimus has been shown to preserve microcirculation after I/R by suppressing the endothelial expression of a powerful vasoconstrictor agent, endothelin (ET)-1 [108]. It also acts on the NO signaling pathway, as it inhibits inducible nitric oxide synthase (iNOS) gene expression by blocking NF-κB activity [109,110,111]. Tacrolimus administered before ischemia has also been shown to maintain the intramitochondrial Ca^2+^ concentration, which preserves mitochondrial function [112,113].

Meanwhile, the close interaction between vascular endothelial cells and circulating inflammatory cells is an important factor in I/R pathophysiology. Tacrolimus attenuates the inflammatory damage that occurs after ischemic organ reperfusion. It reduces the expression of the adhesion molecules P-selectin and intercellular adhesion molecule 1 (ICAM-1), resulting in low leukocyte rolling and adhesion to vascular surfaces under shear flow [114]. It has also been shown that tacrolimus blocks NF-κB activation and thus decreases ICAM-1 gene transcription [115]. Other studies have shown that pretreatment with tacrolimus is associated with a decline in the expression of interferon-γ (IFN-γ) and TNFα [104,115,116,117,118].

Based on these results, tacrolimus has been studied as an additive in a graft rinse solution [119]. Indeed, flushing a liver graft before transplantation with a solution containing tacrolimus has been shown to cause increased early graft function and decreased hepatocellular injury after reperfusion, compared with flushing with a placebo.

### 3.3. Carvedilol

Carvedilol, [1-[carbazolyl-(4)-oxy]-3-[(2-methoxyphenoxyethyl)amino]-2-propanol], is a nonselective β-adrenergic antagonist, which exerts a vasodilatory effect via the blockage of α1-adrenergic receptors [120]. Apart from this direct vasodilatory action, which favors the distribution of preservation solutions throughout an organ, cooling it uniformly, carvedilol has ROS scavenging properties [121], inhibits the adhesion and activation of PNNs and protects endothelial function. This drug therefore seems to be a good candidate to reduce the effects of I/R. Indeed, carvedilol and even some of its metabolites have antioxidant effects [122,123,124,125].

Carvedilol-induced inhibition of lipid peroxidation has been attributed to its capacity to sequester Fe^3+^ ions [122,126]. Moreover, the antioxidant activity of carvedilol has been found to be about ten times that of vitamin E, and several metabolites of carvedilol are more potent than the parent drug [127,128,129,130,131]. In addition, carvedilol has been shown to maintain the activity of endogenous antioxidant systems and to scavenge O^2−^ anions and OH·radicals from both aqueous media and membrane lipids after reperfusion of ischemic myocardium [123,124,130,132]. Meanwhile, it is known that oxidative stress can cause not only direct cytotoxic effects, but also events resulting in the modulation of gene expression associated with polynuclear neutrophil (PNN) activation and the establishment of inflammation and tissue remodeling. Carvedilol has been found to decrease the infiltration of PNN [133] through the inhibition of its attachment to cell membranes and the reduction of endothelial cell activation [134]. A recent study also reported that carvedilol reduced inflammation in a hepatic I/R model [135].

Carvedilol improves Ca^2+^ homeostasis by modulating Ca^2+^ intracellular concentration independently of phospholipase-C (PLC), thus avoiding mitochondrial dysfunction [136,137]. In a rabbit myocardial I/R model, it was found that carvedilol reduces the number of apoptotic myocytes in ischemic areas by 77%. This effect was attributed to the inhibition of Fas protein upregulation and stress-activated protein kinase (SAPK) activation by ROS in the myocardium [138]. Moreover, it has been reported that carvedilol can effectively prevent apoptosis by modulating the mitogen-activated protein (MAP) kinase and caspase-3 signal transduction pathway [139,140,141]. Further studies using other β-adrenergic antagonists, such as atenolol or propranolol, have demonstrated the attenuation of myocardial cell or endothelial cell apoptosis, but these protective effects of the drugs were significantly weaker than those of carvedilol [138,139]. Carvedilol was shown to decrease apoptosis by modulating various pro/antiapoptotic pathways and caspases, in different in vivo and in vitro models [142]. Indeed, it inhibits proapoptotic pathways, such as the caspase-3 and caspase-9 pathways, thus reducing cell death. In addition, it promotes the activation of the antiapoptotic caspase Bcl-2 [142]. 

Several studies have indicated that carvedilol improves endothelial function [143]. Indeed, comparing the effects of metoprolol and carvedilol, Bank et al. found no difference between the two compounds in terms of oxidative stress, but a clear advantage in terms of endothelial function was observed for carvedilol [144]. Carvedilol can also decrease the production of ET-1, the potent vasoconstrictor agent mentioned above. Interestingly, this was not the case for other β-adrenergic antagonists, such as propranolol or cicloprolol, and the action was not blocked by the inhibition of NO synthase by L-NAME (Nγ-nitro-l-arginine methyl ester) [145]. Under ischemic conditions, carvedilol can activate AMP kinase in cardiomyocytes as well as in perfused mouse hearts [136].

Carvedilol, used as an additive to a preservation solution, is of interest due its potential to protect against IRI during transplantation. It has been widely reported that AMPK activation promotes NO synthesis, which in turn protects steatotic and nonsteatotic livers against IRI. Ben Mosbah et al. demonstrated a protective action of carvedilol when added to a UW solution [125]; they found a decrease in transaminases, vascular resistance and oxidative stress. In parallel, they also observed an augmentation of hepatic clearance of bromosulfophthalein, preservation of mitochondria and tissue ATP production and activation of AMPK and eNOS. These results were observed for both steatotic and nonsteatotic livers, opening up a promising alternative for these organs, the latter of which are even more sensitive to IRI.

**Figure 2:**
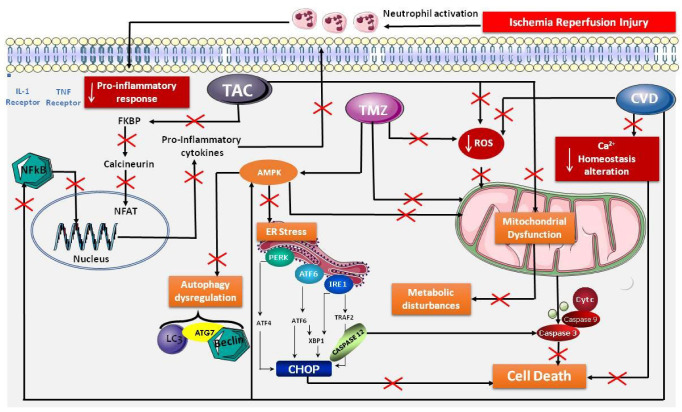
Schematic representation of the newly proposed signaling pathways of the specified drugs (trimetazidine, tacrolimus and carvedilol) in organ preservation aimed at regulating the mechanisms responsible for IRI. ↓: decrease; ↑: increase; AMPK: AMP-activated protein kinase; ATG7: autophagy-related gene 7; ATP: adenosine triphosphate; Ca^2+^: calcium ion; CHOP: C/EBP homologous protein; CVD: carvedilol; ER: endoplasmic reticulum; FKBP: FK506-binding protein; LC3B: light chain 3 B; NF-AT: nuclear factor of activated T-cells; NF-κB: nuclear factor kappa-light-chain-enhancer of activated B cells; NO: nitric oxide; PERK: protein kinase RNA-like ER kinase; ROS: reactive oxygen species; TAC: tacrolimus; TMZ: trimetazidine.

On the basis of the cited literature, these three molecules might offer a new strategy, and they may be capable of acting simultaneously, thereby regulating different targets responsible for IRI and thus protecting the organs. Figure 2 is a representation of the signaling pathways we propose for trimetazidine, carvedilol and tacrolimus, in protection against IRI.

## 4. Conclusions

Transplantation is a complex procedure that requires optimal conditions to ensure the long-term survival of the graft. The inevitable storage period between removal of the organ from the donor up to transplantation to the recipient generates an IRI.

Current hypothermic solutions have their advantages and limitations. However, they all contain the same protective agents, which have not proven to be effective in protecting against IRI.

Moreover, the increase in organs from extended criteria donors, which are more sensitive to IRI, combined with a better understanding of impacts of IRI, calls for an even better attention to preservation.

Thus, the current main goal in the field of organ preservation is to define a formulation that includes new stable pharmacological components that protect against IRI without involving additional clinical risks.

Trimetazidine, carvedilol and tacrolimus, which are safe multitarget drugs, seem to be good candidates for inclusion in the formulation of a new improved solution; they could be included into an optimal extracellular-type washing solution base, rich in sodium and with PEG as an oncotic agent.

This strategy is about to move from today’s cold storage solution to tomorrow’s cold preservation solution. It will design a potential route towards optimized organ protection in transplantation.

## Figures and Tables

**Table 1:** Main components of cold storage solutions (CSSs) and machine perfusion solutions (MPSs). CSS: cold storage solution; HES: hydroxyethyl starch; HTK: histidine–tryptophan–ketoglutarate; IRI: ischemia/reperfusion injury; K^+^: potassium intracellular ion; MPS: University of Wisconsin (Belzer) machine perfusion solution; Na^+^: sodium extracellular ion; PEG: polyethylene glycol; UW: University of Wisconsin (Belzer).

Component/Function	Cold Storage Solutions (CSSs)				Machine Perfusion Solutions (MPSs)
	HTK	UW CSSs	Celsior	IGL-1	UW MPSs
Osmotic	Manitol/Ketoglutarate	Raffinose/Lactobionate	Manitol/Lactobionate	Raffinose/Lactobionate	Glucose/Gluconate/Ribose
Buffer	Histidine	PO4	Histidine	PO4	PO4/HEPES
Oncotic	−	HES	−	PEG	HES
Na^+^	Low	Low	High	High	High
K^+^	Low	High	Low	Low	Low
Antioxidant/IRI Protection	Tryptophan	Adenosine/ Glutathione/Allopurinol	Glutathione	Adenosine/Glutathione/ Allopurinol	Glutathione/Adenine

## Data Availability

Not applicable.
